# An improved method for *Agrobacterium rhizogenes*-mediated transformation of tomato suitable for the study of arbuscular mycorrhizal symbiosis

**DOI:** 10.1186/s13007-018-0304-9

**Published:** 2018-05-09

**Authors:** Tania Ho-Plágaro, Raúl Huertas, María I. Tamayo-Navarrete, Juan A. Ocampo, José M. García-Garrido

**Affiliations:** 1 Department of Soil Microbiology and Symbiotic Systems, Estación Experimental del Zaidín (EEZ), CSIC, Calle Profesor Albareda n◦1, 18008 Granada, Spain; 2 Noble Research Institute LLC, 2510 Sam Noble Parkway, Ardmore, OK 73401 USA

**Keywords:** Transformation, Composite plants, Hairy roots, Tomato, Arbuscular mycorrhiza

## Abstract

**Background:**

*Solanum lycopersicum*, an economically important crop grown worldwide, has been used as a model for the study of arbuscular mycorrhizal (AM) symbiosis in non-legume plants for several years and several cDNA array hybridization studies have revealed specific transcriptomic profiles of mycorrhizal tomato roots. However, a method to easily screen candidate genes which could play an important role during tomato mycorrhization is required.

**Results:**

We have developed an optimized procedure for composite tomato plant obtaining achieved through *Agrobacterium rhizogenes*-mediated transformation. This protocol involves the unusual in vitro culture of composite plants between two filter papers placed on the culture media. In addition, we show that *DsRed* is an appropriate molecular marker for the precise selection of cotransformed tomato hairy roots*. S. lycopersicum* composite plant hairy roots appear to be colonized by the AM fungus *Rhizophagus irregularis* in a manner similar to that of normal roots, and a modified construct useful for localizing the expression of promoters putatively associated with mycorrhization was developed and tested.

**Conclusions:**

In this study, we present an easy, fast and low-cost procedure to study AM symbiosis in tomato roots.

## Background

The *Solanum lycopersicum* (tomato) is an economically important crop grown worldwide. Tomato production accounts for 16% of global vegetable production and constitutes the largest vegetable category. The total tomato crop is forecast to reach 38.6 million tons in 2017 and is expected to continue growing (https://www.wptc.to/).

Tomato crops are susceptible to many insects, bacteria and nematodes which cause significant reductions in fruit yields under current production practices [1]. Rhizosphere symbiotic microorganisms are increasingly proposed as a possible alternative to overcoming these problems [2], with particular emphasis on arbuscular mycorrhizal (AM) fungi which form mutualistic symbiotic association with most higher plants. Although tomato growth is not very responsive to AM fungi [3], mycorrhization significantly reduces tomato infection and disease severity caused by several root pathogens [4–7] and is regarded as a potential beneficial crop management tool to reduce pathogen populations in the soil which positively impacts crop health, quality and production [8].

In addition to its cultivation and economic importance worldwide, *S. lycopersicum* has become a convenient model for research into plant genetics and physiology due to several features such as its relatively compact genome (950 Mb), the availability of a marker-saturated genetic linkage map and the annotated genome sequence (http://solgenomics.net), rich germplasm collections (Tomato Genetics Resource Center, http://tgrc.ucdavis.edu) and highly efficient transformation protocols [9].

Several microarray analyses [10–12] have produced specific transcriptomic profiles of mycorrhizal tomato roots. However, the development of a procedure to easily screen candidate genes performing important functions during mycorrhization is required. *Agrobacterium rhizogenes*-mediated transformation offers a fast and reliable alternative to stable transformation when research is focused on root biology. Unlike *A. tumefaciens*, *A. rhizogenes* contains root locus (*rol*) genes, which, on induction, trigger the development of adventitious, genetically transformed so-called hairy roots due to the conspicuous abundance of their root hairs, a term first used in the literature by Stewart et al. [13].

Using *A. rhizogenes*-mediated transformation, healthy composite plants consisting of untransformed shoots with transgenic hairy roots can be generated. Protocols for obtaining composite plants have been proposed in order to easily pinpoint genes resistant to root pathogens in coffee [14], peanuts [15], soybean [16–18], potato [19], and even tree species such as avocado [20] and *Prunus* [21]. These techniques have also been developed to assess genes predicted to function in the plant parasitism of neighboring roots [22] and to study root symbiotic interactions including AM and *Rhizobium* symbiosis in legume species such as soybean [16], pea [23] and *Medicago truncatula* [24–26].

Although some molecular studies have been carried out using in vitro tomato hairy root cultures, e.g. for the study of *Fusarium* tolerance [27] or of changes in cell wall ligno-suberization associated with salt stress [28], no reports exist regarding the use of this system to study AM symbiosis in tomato and, to our knowledge, no specific protocol currently exists for obtaining composite tomato plants in vitro. Only one paper have previously reported the obtaining of composite tomato plants [29], however the protocol was not specific for tomato and the authors did not used in vitro conditions.

The aim of this work was to show the difficulties found for in vitro composite tomato plant obtaining and we set up a method to overcome these problems. The major challenges we encountered were the excessive handling required and the consequent high level of contamination-related loss. The main factor which hampered composite tomato plant obtaining was the specific growth of tomato hairy roots which penetrate deeply into the culture media. In order to assist in tracking the success of the transformation procedure and to distinguish between non-cotransformed and transgene-containing roots, we used DsRed as a visual marker protein that delivers red fluorescence, and we tested that it can be successfully used as a marker to undoubtedly identify and select tomato cotransformed hairy roots from the beginning of the in vitro culture until the harvesting time. In this paper, we present a strategy for fast and efficient production of *S. lycopersicum* composite plants using *A. rhizogenes* MSU440, in which the transgenic roots of the composite plants showed the expression of the red fluorescence protein marker (DsRed). We demonstrated that these plants are efficiently mycorrhized by *Rhizophagus irregularis* and also showed the utility of this method for localizing the expression of AM-induced promoters. The procedure developed would now favor undertaking further research into the molecular mechanisms involved in AM symbiosis in tomato.

## Methods

### Binary vectors and *A. rhizogenes* strain

We selected three different binary vectors to facilitate transgenic overexpression, RNAi silencing and localization of promoter-GUS expression. The three vectors, all of which contained the *DsRed* marker gene for identification and selection of cotransformed hairy roots, used GATEWAY technology (Invitrogen) for transgene and promoter cloning. The following vectors were selected: pUBIcGFP-DR [30] for overexpression, pK7GWIWG2_II-RedRoot (http://gateway.psb.ugent.be) for RNAi silencing and pBGWFS7::pAtUbq10::DsRed for promoter-GUS expression analysis. The latter vector was obtained by cloning the pAtUbq10::DsRed fragment in the pBGWFS7 vector [31] using the In-Fusion HD Cloning Kit (Takara). In order to check the proper functioning of this plasmid, the arbuscular-induced phosphate transporter 4 promoter (p*SlPT4*) was subcloned. The empty binary vectors pUBIcGFP-DR, pK7GWIWG2_II-RedRoot and pBGWFS7::pAtUbq10::DsRed, as well as the construct pBGWFS7:: pAtUbq10::DsRed::p*SlPT4* were introduced into *A. rhizogenes* MSU440 by electroporation, and transformants were selected by resistance to streptomycin and spectinomycin.

### Generation of composite *S. lycopersicum* plants

**Fig. 1:**
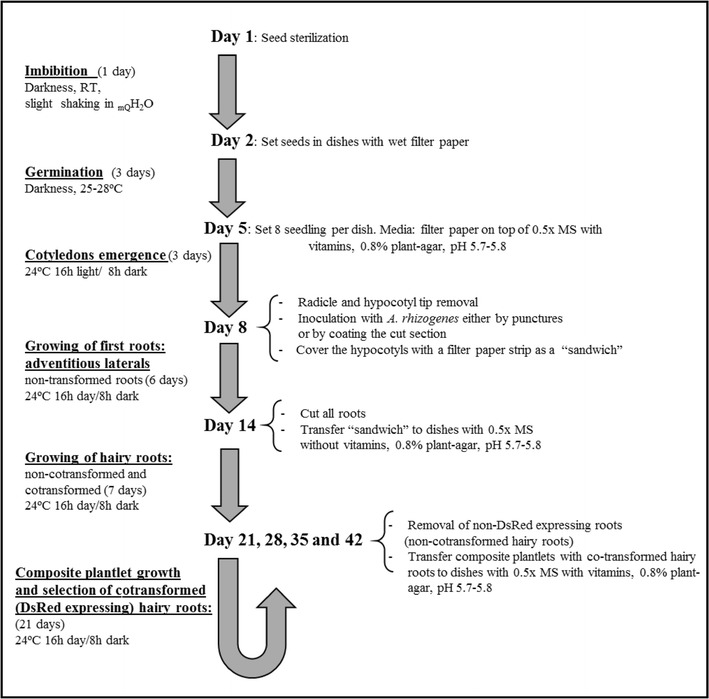
Schematic representation of the time-line required for the generation of composite tomato plants, with an emphasis on the parameters which must be optimized

**Fig. 2:**
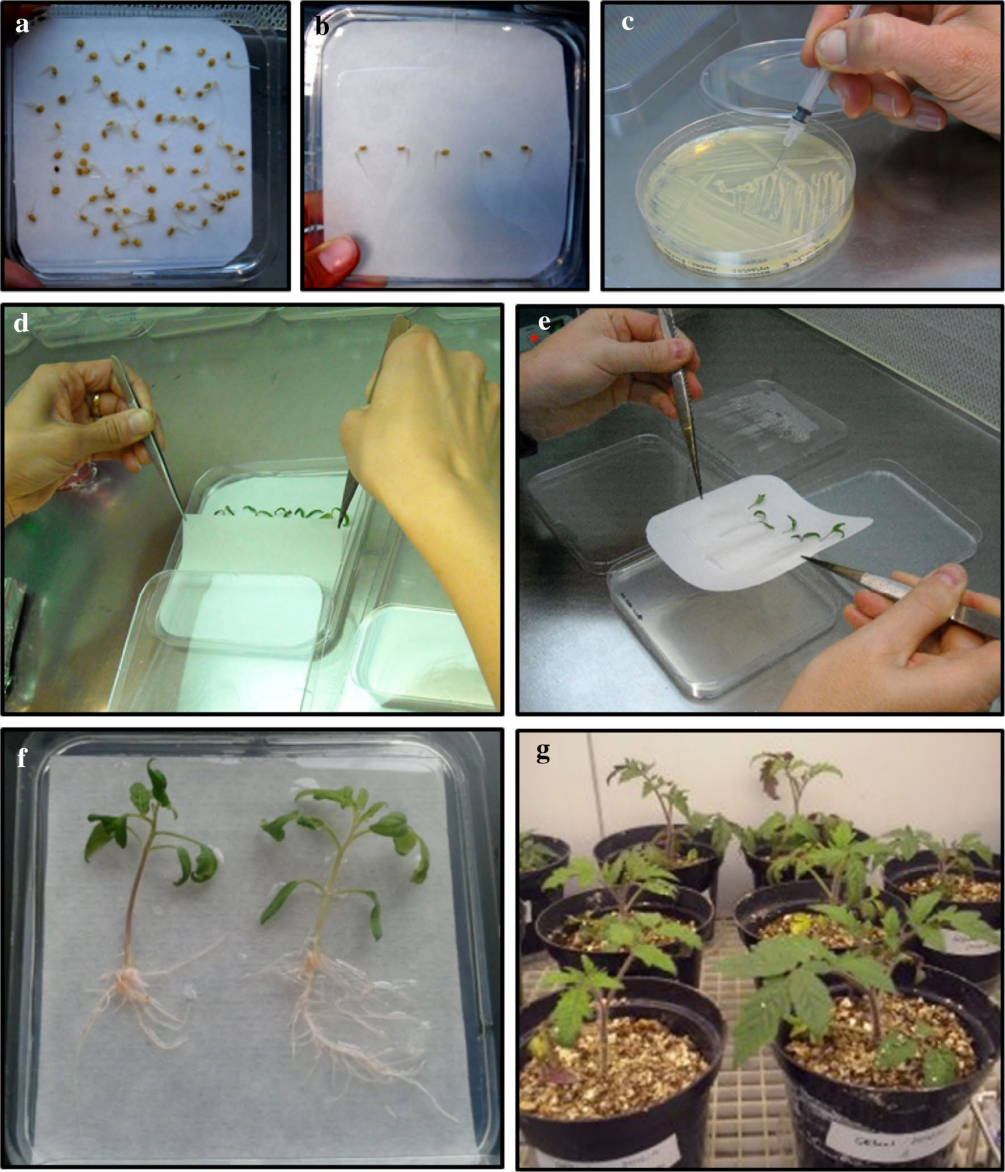
Important steps for tomato composite plant production. Seed germination (**a**) and germinated seeds placed in petri dishes with MS media (**b**). *A. rhizogenes* bacterial culture and syringe used to inoculate tomato hypocotyls by punctures (**c**). Setting of the filter paper strip (**d**) and easy transfer of the plants to new/fresh media using the “sandwich” method (**e**). Resulting vigorous composite tomato plantlets (**f**) and derived composite plants growing in pots (**g**)

Figures 1 and 2 highlight the important steps of the procedure which most easily achieved the highest transformation efficiencies. *A. rhizogenes* MSU440 cultures harboring the corresponding overexpression, RNAi and promoter-GUS constructs were used to transform *S. lycopersicum* plantlets according to a technique adapted from a previously described protocol [26]. *S. lycopersicum* cv Moneymaker plant seeds were surface sterilized by soaking for 5 min using 2.35% w/v sodium hypochloride (50% v/v commercial bleach) and then washed three times with sterile distilled water. In order to improve the germination percentage, speed and uniformity, seeds were subjected to 1-day imbibition in distilled water in the dark at RT with slight shaking. Seeds were then placed and incubated in sterile and sealed Petri dishes with wet filter paper in the dark at 25–28 °C for 4 days. After this period, germinated seeds were placed on filter paper on 0.5 × MS agar medium (0.8% agar) plates which included vitamins to allow for favorable conditions at seedling emergence and during the first days following infection. The germinated seeds were left to emerge in a growth chamber for 4 days at 24 °C, with a 16 h light/8 h dark photoperiod and ∼ 115 μmol m^−2^ s^−1^ of light intensity. To generate hairy roots (genetically transformed), the radicle and the bottom part of the hypocotyl from these 4-day-old seedlings were removed, while maintaining the 1-cm apical portion of the hypocotyl. A sterile syringe tip coated with transgenic *A. rhizogenes* was used to puncture the stem of the cutting 3–4 times. Alternatively, diagonally cut radicles were coated with *A. rhizogenes*. These wounded seedlings were kept on the same 0.5 × MS agar plates, and their hypocotyls were covered with a humid filter paper strip to maintain moisture. The seedlings were left to root in a growth chamber under the same conditions as before.

Roots grown during the first week (adventitious laterals, non-transformed roots) were removed, and plantlets were transferred to 0.5× MS agar medium (0.8% agar) plates without vitamins in order to boost new root development. During the following 3–4 weeks, screening and removal of DsRed-negative roots (non-transformed) were carried out weekly by observation under a fluorescent Leica M165F stereomicroscope. Plantlets with at least one cotransformed hairy root were transferred to 0.5× MS agar medium (0.8% agar) with vitamins. Finally, seedlings with selected DsRed-positive roots (hairy roots) were transplanted to pots and allowed to grow in a growth chamber.

### Plant growth conditions

Composite tomato seedlings obtained in vitro were grown in an autoclave-sterilized (20 min at 120 °C) mixture of expanded clay, washed vermiculite and coconut fiber (2:2:1, by volume). Plant growth and treatments took place in a growth chamber (day: night cycle, 16 h, 24 °C; 8 h, 20 °C; relative humidity 50%). Inoculation with *R. irregularis* (DAOM 197198) was carried out in 500-ml pots as previously described [32]. Each seedling was grown in a separate pot and was inoculated with a piece of monoxenic culture in Gel-Gro medium containing 50 *R. irregularis* spores and infected carrot roots. As hairy root tomato plantlets strongly suffer during transfer from in vitro to pot culture, plastic glasses or humidity domes were placed on top of these plants and sprayed with water every 2 days to maintain moisture and to prevent wilting during the first weeks. One week after planting and weekly thereafter, 20 ml of a modified Long Ashton nutrient solution containing 25% of the standard phosphorus (P) concentration was added to the pots [33], as low phosphate conditions have been shown to stimulate hyphal branching, consequently improving the rate of colonization by the AM fungi [34, 35]. Plants were harvested at different times after inoculation, and the root system was washed and rinsed several times with tap water. DsRed-negative roots (non-transformed laterals or non-cotransformed hairy roots) were removed and screened by observation under a fluorescent Leica M165F stereomicroscope.

### AM fungal staining

A non-vital trypan blue histochemical staining procedure was used [36]. Stained roots were observed with a light microscope, and the intensity of root cortex colonization by the AM fungus was determined as previously described by Trouvelot [37].

### RNA extractions and gene expression quantification

Total RNA was isolated from about 0.2 g-samples using the RNeasy Plant Mini Kit (Qiagen, Hilden, Germany) following the manufacturer’s instructions and treated with RNase-Free DNase. 1 µg of DNAse-treated RNA was reverse-transcribed into cDNA using the iScriptTM cDNA synthesis kit (Bio-Rad, Hercules, CA, USA) following the supplier’s protocol. For qPCR, a 20 μL PCR reaction containing 1 μL of diluted cDNA (1:10), 10 μL 2× SYBR Green Supermix (Bio-Rad, Hercules, CA, USA) and 200 nM of each primer was prepared using a 96-well plate. The experiment was carried out on three biological replicates, and the threshold cycle (Ct) was determined in triplicate. The relative transcription levels were calculated by using the 2^−ΔΔCt^ method [38]. The Ct values of *SlPT4* gene were normalized to the Ct value of the *LeEF*-*1α* (accession number X14449) housekeeping gene. The qPCR data for *SlPT4* gene was shown as relative expression with respect to the control treatment (non-AM inoculated roots) to which an expression value of 1 was assigned. Primers used were 5′-GGTGGCGAGCATGATTTTGA-3′and 5′-CGAGCCAACCATGGAAAACAA-3′ for *LeEF*-*1α*; and 5′-GAAGGGGAGCCATTTAATGTGG-3′ and 5′-ATCGCGGCTTGTTTAGCATTTCC-3′ for *SlPT4* expression analyses.

### Histochemical localization of GUS activity and the AM fungus

AM inoculated and non-inoculated transgenic roots carrying the *SlPT4* promoter-GUS fusion were subjected to GUS staining based on a technique earlier developed by Jefferson [39]. Hairy roots were vacuum-infiltrated with a GUS staining solution composed of 0.05 M sodium phosphate buffer, 1 mM potassium ferrocyanide, 1 mM potassium ferricyanide, 0.05% Triton X-100, 10.6 mM EDTA-Na and 5 μg ml^−1^ X-gluc cyclohexylammonium salt (previously dissolved in *N*,*N*-dimethylformamide) for 30 min to improve substrate penetration. The tissues were then incubated in the dark at 37 °C from 1 h to overnight or until staining was satisfactory in the same staining solution. For co-staining of the AM fungus, GUS-stained hairy roots were embedded in hot (∼ 60 °C) liquid 4% agarose in PBS 1X. 60 μm longitudinal sections were cut on a vibratome (Leica VT1200S) and vacuum-infiltrated with 10 μg ml^−1^ WGA-Alexa Fluor 488 conjugate (Molecular Probes, Eugene, Oreg., USA) in PBS 1X for 20 min in the dark. Cuttings were washed with PBS 1X. A stereomicroscope Leica M165F and an inverted fluorescent microscope (Leica DMI600B) were used for visualization.

## Results

### *rhizogenes*-mediated tomato transformation

**Fig. 3:**
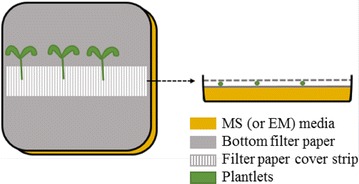
Illustration of the “sandwich” method for in vitro culture of tomato composite plants. Hypocotyl location in between of two filter papers was the best choice found for easy handling and to produce vigorous composite plants

**Fig. 4:**
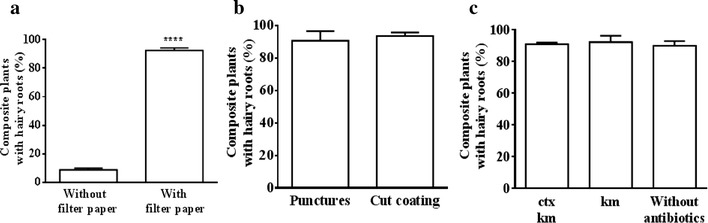
Efficiency of different methods for tomato composite plant production. The percentage of 30 day-old vigorous composite plants grown in pots with respect to the initially *A. rhizogenes* inoculated seedlings is shown. The effectiveness of a filter paper strip covering the wounded *A. rhizogenes* inoculated hypocotyls in sandwich mode was proved (**a**), and we compare two *A. rhizogenes* inoculation methods: by punctures or by coating the bottom part of the hypocotyl with the bacterial culture (**b**). The requirement of cefotaxime (500 μg ml^−1^) and kanamycin (100 μg ml^−1^) antibiotics was tested (**c**). When not specified, standard conditions were used, which include the sandwich mode, *A. rhizogenes* inoculation by punctures and the use of antibiotics. Data corresponds to at least three independent experiments starting from 100 germinated seeds. *A. rhizogenes* carried the empty vectors pK7GWIWG2_II-RedRoot (**a**–**c**), pUBIcGFP-DR (**a**, **b**) or pBGWFS7::pAtUbq10::DsRed (**a**, **b**). Values correspond to mean ± SE. Significant differences (Student’s t test) between treatments are indicated with asterisks (****P < 0.0001)

Most of the in vitro procedures used for generating composite plants involve placing the *A. rhizogenes* inoculated sectioned seedlings on slanted agar [26]. We found that *S. lycopersicum* roots, in contrast to *M. trucatula* roots, deeply penetrated the agar media; it was extremely difficult to remove untransformed adventitious laterals and non-cotransformed hairy roots and to detach the derived composite plantlets from the media without breaking the roots. To cope with this problem, we placed a filter paper on the plate media as previously recommended by certain researchers [22, 40]. After using this method, drying and detrimental effects on shoot growth were observed, and only 26 ± 8% of the composite seedlings could be transferred to pots, many of which subsequently died after transfer to pots. Using this method, we obtained a final survival rate of only 9 ± 1%. We tested a modified method involving the placement of an additional filter paper strip on the hypocotyls in sandwich mode during the whole in vitro culture process as illustrated in Fig. 3. Using this protocol, transformation efficiency was maintained, while seedling drying was totally prevented, and a significant improvement in composite seedling fitness was observed. With this modification, it was possible to transfer and successfully grow 93 ± 2% of the initial transformed seedlings in pots (Fig. 4a).

### *Rhizogenes* inoculation method

The most common inoculation procedure used for generating composite plants involves stab inoculation [41]. However, many authors have found that growth is significantly improved when *A. rhizogenes* is inoculated directly on freshly sectioned seedling radicles or hypocotyls in some species [22, 26, 42]. In order to determine the most appropriate method for tomato transformation, *S. lycopersicum* plants were inoculated with *A. rhizogenes* either by puncturing the seedlings with a glass needle containing bacteria or by coating the fresh diagonally-cut ends of hypocotyls into bacterial cultures. During the first week following inoculation, a small number of non-transformed adventitious laterals appeared just above the hypocotyl section regardless of the *A. rhizogenes* inoculation method used, which is a normal response of tomato seedlings to removal of the root apical meristem. Two weeks after inoculation, puncture inoculation resulted in approximately 50% of the plantlets developing cotransformed hairy roots, while only 25% of the coated-inoculated seedlings had regenerated cotransformed hairy roots by that time. However, 4 weeks after *A. rhizogenes* inoculation and consecutive weekly removal of non-cotransformed hairy roots (non-DsRed expressing roots), at least 90% of plantlets had developed cotransformed hairy roots in both punctured and coated-inoculated seedlings. Although transgenic root growth was boosted by the puncture method, both methods were found to be effective for obtaining composite plants, with the final test showing similar success (Fig. 4b). These results are different to those obtained with *M. truncatula* [26] and *Triphysaria versicolor* [22], where transformation efficiency increased by dipping the freshly sectioned hypocothyls into *Agrobacterium* cultures. We therefore conclude that the effectiveness of the type of wound for *A. rhizogenes* infection is species-dependent.

### Elimination of *A. rhizogenes* is not required

Most protocols proposed for obtaining composite plants include the use of antibiotics, such as timentin, cefotaxime or augmentin depending on the *A. rhizogenes* strain used, in order to inhibit *A. rhizogenes* growth on the media of the in vitro composite plant culture [22, 26]. We noticed that, in the absence of cefotaxime, *A. rhizogenes* did not spread out in the in vitro culture used in this study, in which the filter paper is placed on the MS media and sucrose is lacking, and the percentage of composite plants obtained is not significantly affected (Fig. 4c).

### Molecular markers: Selection for cotransformed hairy roots by DsRed

**Fig. 5:**
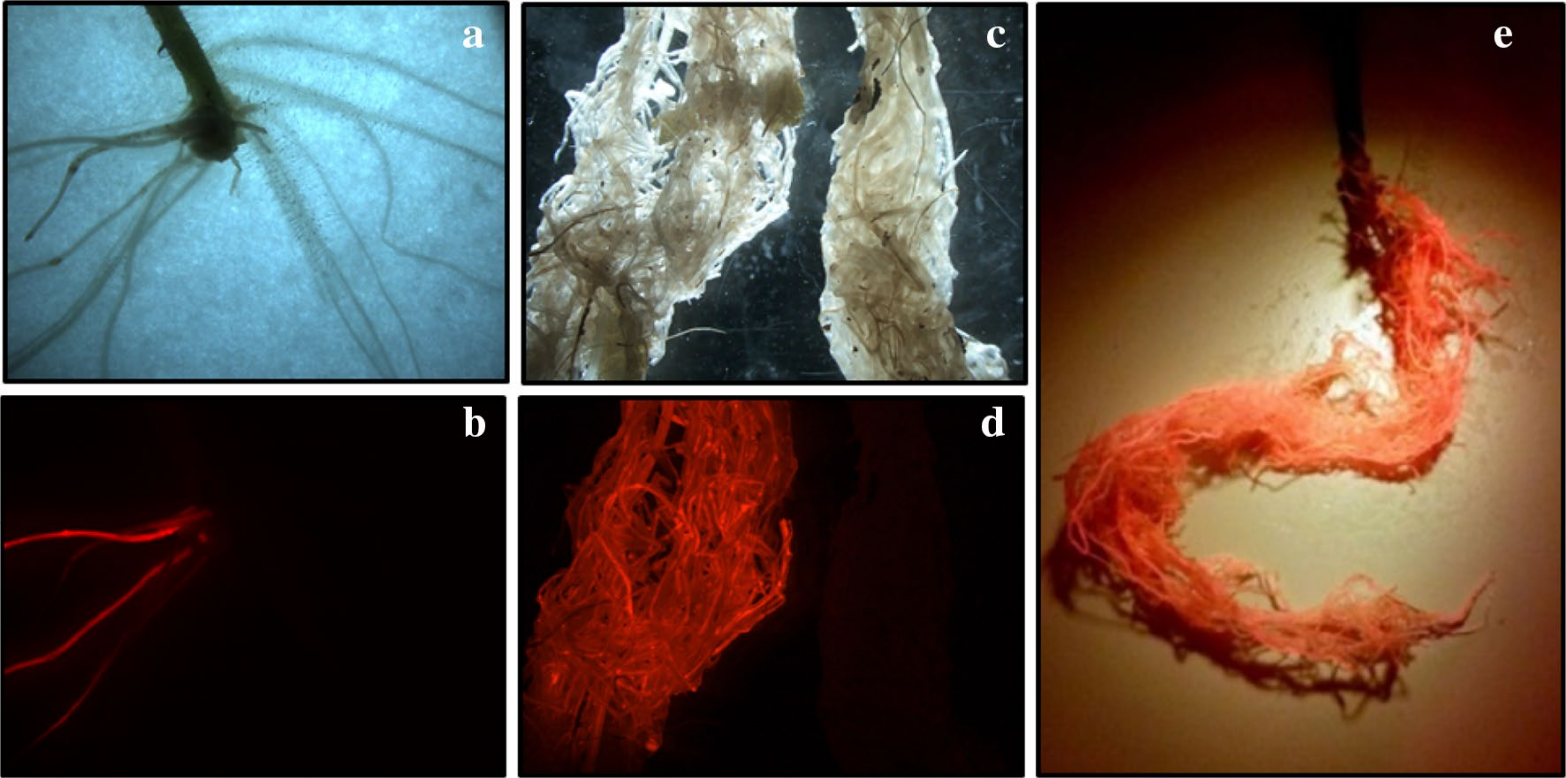
Screening of cotransformed hairy roots expressing the DsRed marker gene in composite tomato plants. Roots of in vitro composite plantlet (**a**, **b**) and composite plants harvested after 45 days growing in a pot (**c**–**e**) were visualized with a stereomicroscope under bright light (**a**, **c**) and under green fluorescence (**b**, **d**) for selection of cotransformed hairy roots. Composite plant exposed to a green fluorescent lamp and visualized through a red–orange photography filter (**e**). Similar results were obtained independently of the binary vector tested: pUBIcGFP-DR, pK7GWIWG2_II-RedRoot or pBGWFS7::pAtUbq10::DsRed

In general, *A. rhizogenes* transformation protocols involve cotransformation, with both the Ri T-DNA and T-DNA of a binary vector containing the transgene of interest together with molecular markers to allow selection of cotransformed hairy roots. We decided to use the *DsRed* gene driven by the *A. thaliana* ubiquitin promoter (p*AtUbq10*) as a molecular marker to select cotransformed hairy roots and to facilitate quantification of transformation efficiency. The excitation wavelength for DsRed range from 554 to 563 nm and the emission wavelength range from 582 to 592 nm. We demonstrated that inspection and selection through visual observation of DsRed expression under a green fluorescence stereomicroscope fully ensure selection of cotransformed hairy roots. Red auto-fluorescence of tomato roots did not cause problems for interpreting the fluorescence, even in young composite plantlets (Fig. 5a, b), as auto-fluorescence was very weak compared to the fluorescence signal from DsRed. The unmistaken appearance of *DsRed* overexpressing roots compared to DsRed negative roots in adult plants was also observed (Fig. 5c, d). If a fluorescent stereomicroscope is unavailable, root exposure to a green LED bulb (492–577 nm wavelength) and visualization through a red–orange photography filter (~ 585 nm) which blocks UV, blue, yellow and green light but passes longer wavelengths, can also be used (Fig. 5e).

Antibiotic counterselection is often used to increase the percentage of hairy roots cotransformed with the binary vector T-DNA and to inhibit growth of non-transformed adventitious laterals and non-cotransfromed hairy roots. After testing a range of kanamycin (Km) concentrations for the growth of hairy roots cotransformed with the plasmid pK7GWIWG2_II-RedRoot, we found that 100 μg ml^−1^ Km was insufficient to totally inhibit non-transformed hairy root initiation and, over this level of concentration, Km began to negatively affect the shoot growth of composite plants. It was not possible to set an optimal concentration for complete inhibition of non-transformed root development without interfering with shoot growth. In order to determine whether additional antibiotic selection is appropriate in order to enhance cotransformed hairy root growth, experimental trials were carried out both with and without kanamycin (100 μg ml^−1^). The number of plantlets, which developed uncotransformed hairy roots, increased by 30% in the absence of Km as compared to those in the presence of Km (100 μg ml^−1^) 2 weeks after infection. However, once they had developed more than two cotransformed hairy roots, regardless of the presence or absence of the antibiotic, after removal of non-cotransformed hairy roots, the composite seedlings rarely developed new untransformed hairy roots and achieved similar final success rates (Fig. 4c). These results show that, although antibiotic selection slightly boosts the growth of cotransformed hairy roots initially, the presence of the antibiotic does not totally eliminate untransformed root development and does not implies a superior efficiency of the method. We show that *S. lycopersicum* transformed roots with pK7GWIWG2_II-RedRoot, pUBIcGFP-DR and the new plasmid pBGWFS7,0::pUbq::DsRed developed in this study are highly suitable for tomato hairy roots obtaining and selection without requiring selective co-transformation antibiotics.

### Transfer of composite tomato plantlets to pots

One of the critical steps of the procedure involves the transfer of composite plantlets from in vitro conditions to pots. In this step, most of the plantlets were not sufficiently strong to acclimate to the new *ex vitro* conditions and died after a few days. We performed a trial for pre-acclimatization and growth of composite plants before transferring to pots, in which the aerial part was exposed to *ex vitro* conditions, while keeping the root system in vitro as explained as follows. A hole was melted in the side and the lid of the Petri dish containing the 0.5x MS medium (0.8% agar) using a hot metal rod. The obtained 4 week old composite tomato plantlets were transferred (one plantlet in each Petri plate), with the roots placed on the surface of the medium and the shoot extending beyond the hole. The gap around shoot emerging from the hole of the Petri dish was sealed with silicone grease. Petri dishes were placed vertically in a growth chamber. In this way, larger and stronger composite plants pre-acclimated to *ex vitro* conditions were obtained. However, this procedure was discarded because it greatly increased contamination of the media. Alternatively, composite plantlets were directly transferred from in vitro conditions to pots, and immediately covered with translucent plastic or fitted with a humidity chamber to maintain humidity for the first 3 weeks, which resulted in survival rates of virtually 100% for the plantlets transferred.

### Symbiotic phenotype and molecular characterization of AM composite plants

**Fig. 6:**
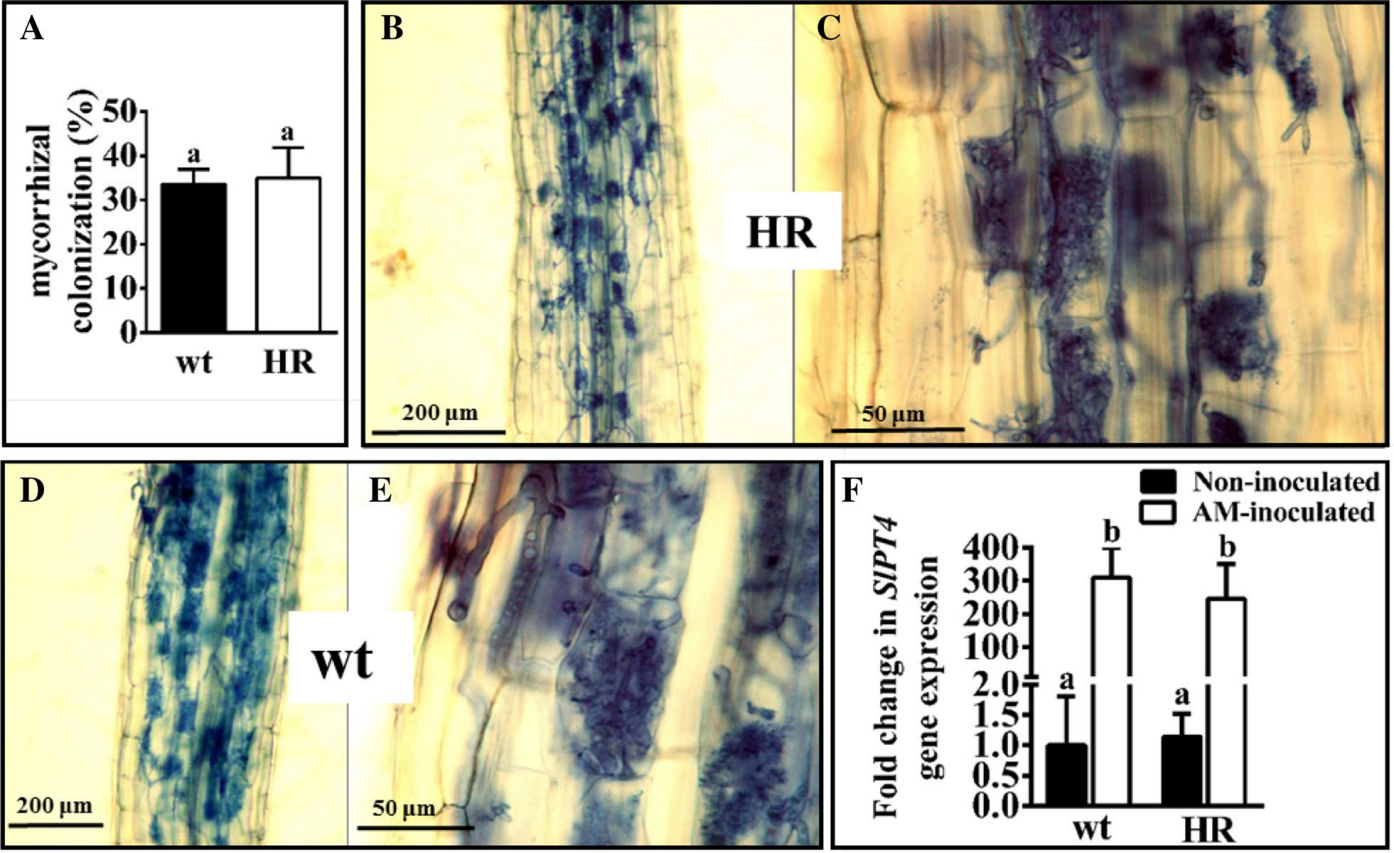
Phenotype and molecular characterization of tomato AM-composite plant roots. Percentage of total root length colonized in wt (adventitious laterals and non-cotranformed hairy roots) and HR (cotransformed hairy roots) from composite plants 45 days after inoculation with *R. irregularis* (**A**). Trypan blue staining showing AM fungal colonization and arbuscule morphology in HR (**B**, **C**) and wt roots (**D**, **E**). Expression of the AM associated gene *SlPT4* in the corresponding roots with respect to the non-inoculated roots (**F**). Values correspond to mean ± SE. Bars with a same letter are not significantly different (P > 0.05) according to LSD multiple comparison test

AM development in *S. lycopersicum* cotransformed hairy roots was studied. Composite plantlets cotransformed with the pK7GWIWG2_II-RedRoot vector were transferred to pots and inoculated with the AM fungus *R. irregularis*. Composite plants were starved of exogenous phosphate to promote mycorrhization. Trypan blue staining revealed that tomato roots began to be colonized by *R. irregularis* 15 days after AM inoculation, that mature arbuscules were present in some areas of the root at 30 dpi and that hairy roots reached roughly 35% mycorrhization at 45 dpi. We did not find any abnormalities in the mycorrhizal phenotype. Apparently, the same mycorrhizal rates (Fig. 6A) and arbuscular morphology were observed in cotransformed hairy roots (Fig. 6B, C) with respect to non-cotransformed hairy roots and adventitious laterals (Fig. 6D, E). In order to confirm the correct functionality of mycorrhization by *R. irregularis* in composite tomato plants, mycorrhizal activity was checked at the molecular level. The *S. lycopersicum SlPT4* marker gene previously described as associated with the mycorrhization process [43, 44] was selected. A similar induction of *SlPT4* was found in both types of AM-inoculated roots (non-cotransformed and cotransformed) with respect to the uninoculated roots, indicating normal mycorrhiza-associated activity in the cotransformed hairy roots (Fig. 6F). These results suggest that transgenic hairy roots can be used to study AM symbiosis.

### Promoter-GUS analysis of AM-composite tomato plants

**Fig. 7:**
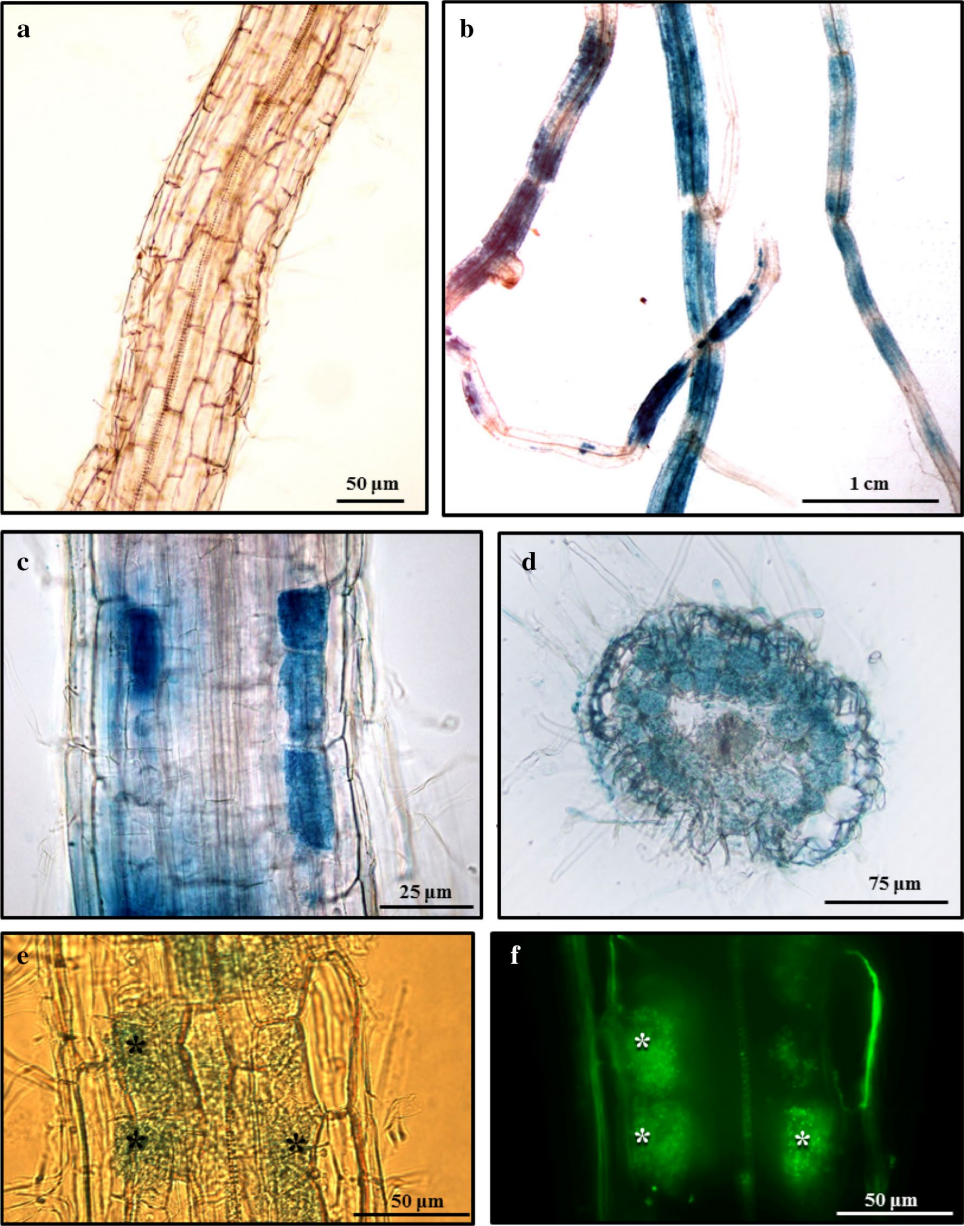
Expression analysis of *SlPT4* promoter activity in cotransformed mycorrhized tomato hairy roots after GUS staining. GUS activity in *A. rhizogenes*-transformed roots expressing the *SlPT4* promoter β-glucuronidase fusion was assessed 45 days after inoculation with *R. irregularis* (**b**–**d**), and without *R. irregularis* (**a**). GUS stained roots were vibratome sectioned (**d**–**f**) and the AM fungus was counterstained with WGA-Alexa Fluor488 to visualize fungal structures (**e**, **f**). Root cells with GUS activity (*SlPT4* promoter expression marker) observed under bright-field (**e**) are coincident to cells containing arbuscules, which deliver green fluorescence due to AM hyphae staining with WGA-Alexa Fluor488 (**f**). Asterisks indicate arbuscules in the host cortical cells. All images were taken with an inverted fluorescent microscope, except in **b**, which was taken with a stereomicroscope

The pBGWFS7,0 plasmid [31] used to localize promoter expression was modified in this study to include *DsRed* marker gene overexpression. In order to test the usefulness of the resulting vector (pBGWFS7,0::pUbq::DsRed) for promoter expression studies of AM fungal colonized tomato hairy roots, we chose the phosphate transporter 4 promoter (p*SlPT4*), which is demonstrated to have an induced expression associated with AM arbuscules [45]. We obtained in vitro composite tomato plantlets with hairy roots cotransformed with the pBGWFS7,0::pAtUbq::DsRed::p*SlPT4* plasmid, which were subsequently AM-inoculated with *R. irregularis* and transferred to pots. 50-dpi cotransformed hairy roots were identified by DsRed fluorescence and collected. As expected, GUS staining was absent in the non-inoculated plants (Fig. 7a) and apparently limited to arbuscule containing cells (Fig. 7b, c), which are located in the root cortex as observed in the cross-sections (Fig. 7d). Counterstaining with WGA-alexa 488 to visualize fungal structures corroborated that p*SlPT4* induction is clearly associated with arbuscules, as shown in Fig. 7e. The results confirm that the pBGWFS7,0::pUbq::DsRed plasmid can be used for *A. rhizogenes*-mediated transformation, DsRed detection of cotransformed hairy roots and promoter-GUS expression analysis of AM roots.

## Discussion

In the protocol developed in this work, we firstly provide experimental evidence that slanted culture of seedlings is not required. Alternatively, the placing of seedlings between two filter papers in sandwich mode is also successful and useful for the composite seedling culture of species such as tomato, whose roots tend to penetrate the culture media. In addition, the tedious task of detaching the agar from roots when transferring plantlets to pots is avoided by using this method.

The other major challenge we overcame in the in vitro culture of composite plants was the frequent contamination of the culture media. Weekly transfer of the plantlets to maintain fresh media and antibiotic activity, as well as taking out the aerial part of the plant greatly increased contamination of the culture media. Procedure modifications introduced, driven to reduce changes in the culture media and to minimize composite plant handling, virtually reduced in vitro culture contamination to zero and simplified the method considerably.

In view of our results, we conclude that large amounts of composite tomato plants can be obtained by using this low-cost and user-friendly handling method, which avoids the use of antibiotics and the tedious task of detaching agar from the roots, and also minimizes the transfer of plantlets to different media and plant manipulation, thus reducing contamination to virtually zero and achieving success rates of 90% and almost 100% at times. In addition, selection of cotransformed tomato hairy roots is completely ensured through the use of the fluorescent molecular marker DsRed. This method provides an improved tool to rapidly assess gene functions in tomato roots without the need for stable transformation plant production. In particular, we showed that the proposed technique is extremely useful for mycorrhizal studies and screening of candidate genes for involvement in AM symbiosis. However, it is important to note that this method might also be suitable for molecular studies of root pathogen interactions affecting tomato, which is an extremely important agricultural species.
